# eHealth Program to Empower Patients in Returning to Normal Activities and Work After Gynecological Surgery: Intervention Mapping as a Useful Method for Development

**DOI:** 10.2196/jmir.1915

**Published:** 2012-10-19

**Authors:** Antonie Vonk Noordegraaf, Judith A.F Huirne, Carina A Pittens, Willem van Mechelen, Jacqueline E.W Broerse, Hans A.M Brölmann, Johannes R Anema

**Affiliations:** ^1^VU University Medical CenterDepartment of Obstetrics and GynaecologyAmsterdamNetherlands; ^2^VU UniversityAthena Institute for Research on Innovation and Communication in Health and Life SciencesAmsterdamNetherlands; ^3^VU University Medical CenterDepartment of Public and Occupational HealthAmsterdamNetherlands

**Keywords:** eHealth intervention, interactive website, Internet, patient empowerment, intervention mapping, gynecological surgery, hysterectomy, laparoscopic adnexal surgery, return to work

## Abstract

**Background:**

Full recovery after gynecological surgery takes much longer than expected regardless of surgical technique or the level of invasiveness. After discharge, detailed convalescence recommendations are not provided to patients typically, and postoperative care is fragmented, poorly coordinated, and given only on demand. For patients, this contributes to irrational beliefs and avoidance of resumption of activities and can result in a prolonged sick leave.

**Objective:**

To develop an eHealth intervention that empowers gynecological patients during the perioperative period to obtain timely return to work (RTW) and prevent work disability.

**Methods:**

The intervention mapping (IM) protocol was used to develop the eHealth intervention. A literature search about behavioral and environmental conditions of prolonged sick leave and delayed RTW in patients was performed. Patients’ needs, attitudes, and beliefs regarding postoperative recovery and resumption of work were identified through focus group discussions. Additionally, a literature search was performed to obtain determinants, methods, and strategies for the development of a suitable interactive eHealth intervention to empower patients to return to normal activities after gynecological surgery, including work. Finally, the eHealth intervention was evaluated by focus group participants, medical doctors, and eHealth specialists through questionnaires.

**Results:**

Twenty-one patients participated in the focus group discussions. Sufficient, uniform, and tailored information regarding surgical procedures, complications, and resumption of activities and work were considered most essential. Knowing who to contact in case of mental or physical complaints, and counseling and tools for work reintegration were also considered important. Finally, opportunities to exchange experiences with other patients were a major issue. Considering the determinants of the Attitude–Social influence–self-Efficacy (ASE) model, various strategies based on a combination of theory and evidence were used, resulting in an eHealth intervention with different interactive functionalities including tailored convalescence recommendations and a video to communicate the most common pitfalls during the perioperative period to patients and employers. Fifteen patients in the focus groups, 11 physicians, and 3 eHealth specialists suggested points for improvement to optimize the usability of the eHealth intervention and judged it an approachable, appropriate, and attractive eHealth intervention to empower gynecological patients.

**Conclusions:**

The IM protocol was a useful method to develop an eHealth intervention based on both theory and evidence. All patients and stakeholders judged the eHealth intervention to be a promising tool to empower gynecological patients during the perioperative period and to help them to return to normal activities and work.

## Introduction

Following gynecological surgery, full recovery (including returning to work) takes much longer than expected regardless of surgical technique or the level of invasiveness [1-3]. In two prospective observational studies in the Netherlands, median sick leave after gynecological procedures for benign conditions was 8 weeks [4]. Prolonged absence from work often results in a lack of social structure and meaningful activities [5,6] and can result in work disability, poorer general health, and increased risk of mental health problems [7,8]. As a result, long periods of sick leave contribute to a reduced quality of life and induce unnecessary yet substantial costs for society through lost working hours, physician consultations, medication treatment, and higher hospital admission rates [9,10].

To reduce health care costs, there is an increasing trend to limit the duration of in-hospital care and to transfer postoperative care to outpatient and primary care [11-13]. However, after discharge, gynecological care is given only on demand, detailed recommendations about resumption of work activities are not provided typically [1,14], and patients often do not know who to contact for support in case of postoperative complaints. Furthermore, family physicians frequently do not give advice about resumption of work activities and occupational or insurance physicians are only consulted if patients have paid work and these consultations take place relatively late in the course of sick leave because of legislation [15-17]. This contributes to irrational beliefs and avoidance of resumption of activities that can result in a prolonged sick leave [18].

Because a significant part of the recovery and return to work (RTW) problems of patients seem to be caused by counseling and communication deficiencies, the starting point of this study was to identify these specific problems. Many interventions aiming to improve communication with and counseling of patients have focused only on health care professionals [19,20]. However, to improve communication and health outcomes, empowering patients to actively participate in their consultations with physicians is also important [21,22]. Patient empowerment refers to the enhanced ability of patients to actively understand and influence their own health status [23]. It focuses on control in patients’ experiences of health, disease, and illness, as well as the roles of health care organizations, communities, and the broader health care system [24,25]. eHealth interventions seem to be a promising way to empower patients by providing personalized education (eg, detailed recommendations on resumption of work activities) and enhancing interaction between health consumers and professionals [26-28]. Patients become more actively engaged in their own state of health (eg, are aware which complications need additional consultation) and the communication between patient and health care provider becomes more efficient and equal [29-31]. Tailored eHealth interventions are more intensively used [32,33] and have a greater impact on people’s behavior [33-36] than generic materials, and they provide the opportunity to deliver information to a large audience [37] at any time and with lower costs [34,38]. An important condition for a successful eHealth intervention is adequate implementation [39,40].

Therefore, the objective of this study was to develop a feasible and generally accepted eHealth intervention that empowers gynecological patients during the perioperative period about returning to normal activities and work, to obtain timely RTW, and prevent work disability. To develop this intervention, we used the intervention mapping (IM) protocol [41,42], which has been shown to be a suitable systematic and scientifically accepted method for the development and implementation of a wide range of eHealth [43-46] and RTW [47,48] interventions based on theory and stakeholders’ (including patients’) involvement.

## Methods

**Figure 1:**
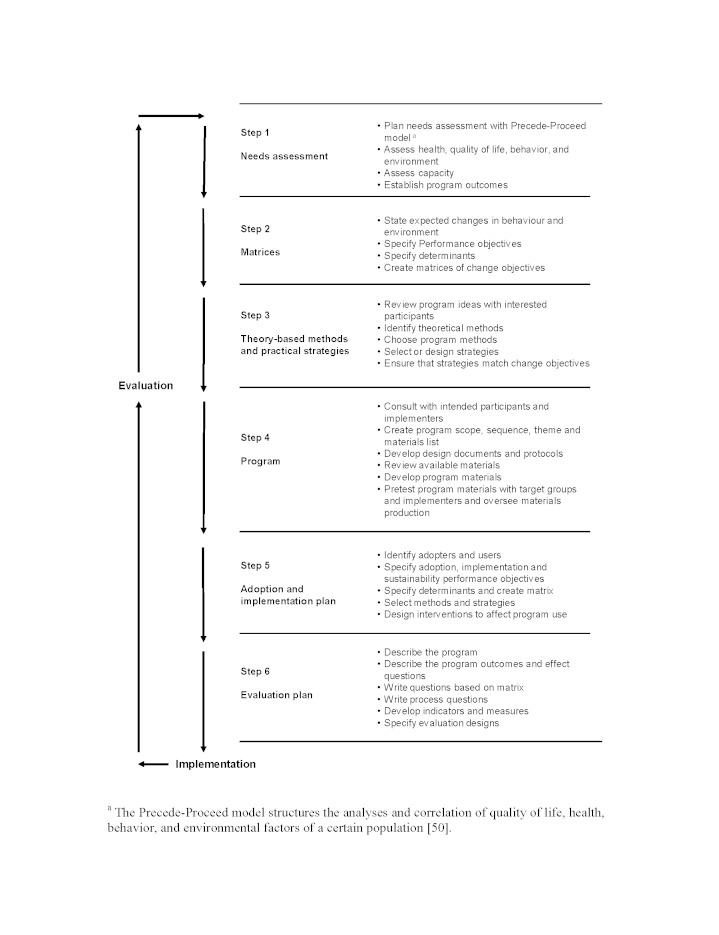
Intervention mapping process.

Intervention mapping was used to tailor the eHealth intervention to patients’ needs and wishes, taking into account the clinical evidence of the main determinants that influence patients’ behavior to reach timely RTW. The project group consisted of 1 research physician, 2 gynecologists, and 2 occupational physicians. Although it is not a theoretical or conceptual framework, IM is a systematic description of a logical planning process involving 6 steps: (1) performing a needs assessment; (2) defining program objectives; (3) selection of theory-based methods and practical strategies; (4) design of the intervention program; (5) development of a plan for adoption and implementation; and (6) design of an evaluation plan (Figure 1). The iterative character of IM enables the intervention to be based on a combination of theory and evidence, which maximizes the applicability for the target population and minimizes the risk of choosing the wrong theory behind the intervention (theory failure) or of poor adoption of the intervention (program failure) [49].

### Step 1: Needs Assessment

In needs assessment, the discrepancy between the current and the desired situation in a given group of people is studied. The needs assessment was structured by the Precede-Proceed model (PRECEDE: predisposing, reinforcing, and enabling constructs in educational diagnosis and evaluation; PROCEED: policy, regulatory, and organizational constructs in educational and environmental development), which analyzes and correlates quality of life, health, behavior, and environmental factors in a certain population [50]. The current situation has shown a large discrepancy between expected duration of physical recovery and actual RTW after gynecological surgery (even laparoscopic), whereas there is strong evidence that long periods of sick leave can result in poorer general health, increased risk of mental health problems and work disability, and induces unnecessary costs for society [2,8] The most frequently performed gynecological surgical procedures with a considerable postoperative effect on recovery and RTW (accounting for more than 17,500 procedures in the Netherlands per year) are hysterectomy (abdominal, vaginal, and laparoscopic) or laparoscopic adnexal surgery on benign indication [51]. Because approximately 67% of women aged 25-65 are in the workforce, these large numbers of surgical procedures have a great impact on absenteeism [52]. Therefore, patients who underwent these types of surgical procedures were chosen as the target group for this intervention.

To clarify and find possible explanations for prolonged sick leave, a literature search in PubMed regarding behavioral and environmental conditions of prolonged sick leave and delayed RTW in the target group was performed.

The focus group technique was considered the most suitable supplement to the literature search for identification of patient’s needs, attitudes, and beliefs regarding postoperative recovery and resumption of work. In addition to supplementing the results of the literature search, it was assumed that focus group discussions would align the results to the Dutch context and give more insight into specific content requirements of the prospective eHealth intervention that could be used during the development process. The participatory technique of focus group discussions is widely used and scientifically accepted to gain insight into public views and needs through group interaction [53,54].

Participants for the focus group discussions were recruited from the patient files of the VU University Medical Center, an academic hospital in the Netherlands. To mirror the intended target group, inclusion criteria for participation in the focus group discussions were age 18-65, a history of a laparoscopic adnexal surgery and/or hysterectomy on benign indication in 2008, and a job (paid or unpaid) of at least 8 hours per week. To create homogeneity within the focus groups but heterogeneity among the groups, the patients were recruited by means of purpose sampling into 3 groups: fast RTW, intermediate RTW, and delayed RTW. All of the participants had already returned to work after surgery (range 1-36 weeks).

The aim of the focus group discussions was to identify patients’ needs regarding perioperative care and counseling in resuming normal and work activities. In addition, patients were specifically asked for the important requirements of a useful eHealth intervention. The identification of patients’ needs and requirements occurred in 3 different steps:

1. Identifying and prioritizing patients’ perceived shortcomings in and difficulties with received perioperative care and counseling in resumption of normal and work activities.

2. Inventory of possible solutions and improvements to overcome these shortcomings and difficulties, starting with the highest prioritized bottlenecks.

3. Brainstorming about favorable content, requirements, and specific tools that should be incorporated into an eHealth intervention that aims to empower patients during the perioperative period and resumption of work activities.

The focus group discussions were all recorded and transferred into verbatim transcripts that were analyzed by open coding using the ATLAS.ti software [55].

A detailed process evaluation of the focus group discussions will be published in a separate paper [Pittens et al, unpublished data, 2012]. The study design and procedures of the focus group discussions were approved by the Medical Ethics Committee of the VU University Medical Center (2009/42, February 9, 2009). Participants signed a privacy agreement to declare voluntary participation, to give permission for processing the information for the development of an intervention (such as an eHealth intervention), and to exclude transmittal of information to others.

As a starting point for the development of the intervention, the products of this first step were the main behavioral and environmental conditions of the chosen target group contributing to prolonged sick leave.

### Step 2: Matrices

The purpose of this step was to transform the identified behaviors and environmental conditions causing prolonged sick leave into behaviors and conditions that prevent a prolonged sick leave. To achieve this, performance objectives were formulated. Performance objectives describe in detail patients’ behavioral and environmental outcomes that are necessary for patients to reach the formulated behavior objective of “timely RTW.”

To select a suitable theoretical framework to reach the performance objectives, a literature search regarding main determinants of recovery and RTW was performed in PubMed. To elucidate, a suitable theoretical framework provides appropriate determinants that could be influenced to reach the behavior objective. Based on this framework, the performance objectives of the target group were elaborated into matrices with change objectives, explaining how patients and their environment will change as a result of the eHealth intervention to reach the behavior objective.

### Step 3: Theory-based Methods and Practical Strategies

In this step, theoretical methods and practical strategies to address the change objectives were searched for and applied. Research has shown that the effectiveness of interventions to change behavior can be increased by the use of theory-based methods [56]. A theory-based method is a method derived from theory and research that describes a process that influences changes in determinants of behavior and environmental conditions. A practical strategy is a technique for the application of the theory-based method in ways that fit the target group and the context in which the eHealth intervention will be applied. The required theoretical framework, theory-based methods, and translation into practical strategies were determined based on the book that describes the IM approach [42], a literature search in the PubMed database, the focus group discussions, and a brainstorm session of the researchers.

### Step 4: Program Plan and Design of the Intervention

During this step, information obtained in previous steps was translated into specific tailored tools and information to empower gynecological patients by the eHealth intervention. Furthermore, to obtain evidence-based information and instruments necessary to fulfill patients’ needs, additional research was performed.

To verify if the eHealth intervention matched with the main target group and fitted the expectations of gynecologists, family physicians, and occupational physicians, the first concept version was evaluated by focus group participants (n = 21), physicians (n = 22), eHealth specialists (n = 3), and a representative of a patient organization (n = 1) through questionnaires. The eHealth intervention was scored on 8 main areas used to describe how the intervention functions, empowers, and can be modified to provide the best behavior change to obtain timely RTW and prevent work disability. The 8 areas included: appearance, behavior prescriptions, burdens of using the website, content, delivery, message, participation, and assessment and tailoring. Ritterband et al [57] describe these areas in detail. This model is meant to ground Internet intervention research within a scientific framework. The 8 different areas were covered in the evaluation questionnaires with 23 unique open- and close-ended questions (Appendices 4 and 5). In addition, participants were also encouraged to propose recommendations. The results of the evaluation were used to optimize the design and usability of the eHealth intervention, which resulted in the final version.

### Step 5: Design of an Implementation Plan

The focus of Step 5 is adoption of the intervention by the patients and relevant stakeholders, and the development of an implementation plan. With the input of patients and stakeholders during previous steps, the researchers identified facilitating factors and barriers regarding adoption and implementation of the eHealth intervention. With this information, an implementation plan to enable an extensive evaluation of the intervention was developed and an appropriate linkage system for future implementation was composed.

### Step 6: Design of an Evaluation Plan

During Step 6, the main objective of this study (ie, to develop a feasible and generally accepted eHealth intervention that empowers gynecological patients during the perioperative period into returning to normal activities and work, to obtain timely RTW, and prevent work disability) was used to compose an evaluation plan. Although the eHealth intervention was based on both theory and evidence and was developed in collaboration with the main target group and relevant stakeholders, its adoption, barriers for usage, and implementation possibilities still had to be evaluated in daily practice. In addition, the effectiveness of this eHealth intervention regarding a timely RTW to prevent work disability had to be investigated. Therefore, the project group approached 7 gynecology practices (1 university-based and 6 hospital-based) about participation in the evaluation of this intervention through implementation of the eHealth intervention as a supplement to the standard perioperative care given at their hospital. In addition, the project group formulated inclusion and exclusion criteria for patients to participate in the study and developed appropriate outcome measures to evaluate the intervention’s effectiveness, adoption, usage, and implementation. Furthermore, a logistic plan to recruit patients and involve participating health care providers was developed.

## Results

### Step 1: Needs Assessment

#### Literature

The literature search revealed that most women extend their sick leave beyond the recommended period on their own initiative [2]. Patients with delays in RTW reported pain/discomfort, feelings of fear, and infections as delaying factors [1]. Those who reported multiple delaying factors reported a variety of combinations that included feelings of fear, anxiety, depression, and differences in employer expectations [1]. Recovery and RTW time is shorter when the patient receives clear and few restrictions that are not too overly cautious at discharge, when the patient has been provided with RTW advice, or when the patient feels urgency to RTW [1,3,58]. Other important environmental conditions for prolonged sick leave and RTW of patients appeared to be the substantial variation in convalescence recommendations given by gynecologists, family physicians, and occupational physicians [58,59]. Their recommendations are not related to the most successful return to normal and RTW activities or the risks of complications [58]. In addition, a lack of clarity regarding absence duration can provide an obstacle for employers and employees who are keen and willing to establish earlier rehabilitation programs, but would not wish to go against the advice of health care providers [59].

#### Focus Group Discussions

Out of 105 invited patients, 38 met our inclusion criteria and were willing to participate in the focus group discussions. On the basis of availability on the selected dates for the focus group discussions, 31 patients were assigned to 3 focus groups. Of these patients, 21 were present at the meetings and participated in the focus group discussions (7 patients per meeting). A process evaluation of the focus group discussions will be published in detail elsewhere [Pittens et al, unpublished data, 2012]*.*

Starting with the first aim of the focus group discussions, the most important reported shortcomings and difficulties of currently provided perioperative gynecological and reintegration care were (in random order):

1. Insufficient or no information about the surgical procedure itself, the logistical process in the hospital from admission to discharge, detailed resumption of work activities after the surgical procedure, and the possible consequences of the surgery (physical and mental).

2. Inconsistency of convalescence recommendations given by gynecologists, family physicians, and occupational physicians.

3. Lack of written instructions on resumption of work activities, tailored to individual conditions and work, and consequently insufficient information and instructions given to relatives.

4. Insecurity with respect to physical or mental postoperative symptoms, complications, or delayed recovery. What to do and whom to contact?

5. Poor communication among gynecologists, family physicians, and occupational physicians resulting in inadequate transfer of information about the procedure and one another’s treatments.

6. Limited or inadequate guidance by occupational physicians because of a lack of knowledge about different types of surgery and corresponding recovery times. Patients reported experiences of occupational physicians forcing the patient to RTW too early or slowing down the RTW process.

7. Difficulties with work reintegration because of insufficient involvement and understanding of the employee/employer during the perioperative and reintegration period.

8. Inability of patients to discuss the perioperative period and reintegration process at work (with employer and colleagues).

9. Lack of a reintegration plan before the surgery.

10. Few opportunities to contact other patients to exchange experiences.

In general, patients mentioned that when they were unsatisfied with the information or counseling given by their doctors and nurses, they asked family and friends who had undergone surgery about their experiences. However, this led to unrealistic expectations because of different types of surgical procedures and techniques, and the fact that recovery is affected by individual conditions and circumstances.

In the second part of the focus group discussions, the patients brought up many possible solutions and improvements to overcome the mentioned shortcomings and difficulties that were processed into performance objectives during Step 2.

Requirements, content, and specific tools that should be incorporated into an eHealth intervention to improve empowerment during the perioperative period may be summarized as follows:

1. Reliable detailed and personalized information about mentioned shortcomings and difficulties in the information supply. Pictures and videos were considered an accessible supplement to transfer this information.

2. Tools for communication with other patients, employers, gynecologists, occupational physicians, and family physicians.

3. Functionalities to develop a personalized reintegration plan.

With the results of the literature search and focus group discussions, the project group concluded that the main determinants of patients’ behavior regarding prolonged sick leave are: (1) inadequate knowledge of important information about the surgery, recovery, and RTW; (2) tendency to extend their sick leave beyond the recommended period; (3) insecurity about postoperative symptoms, complications, and delayed recovery without knowing where to receive appropriate help; (4) lack of skills to compose a work-reintegration plan and to identify possible barriers for RTW; and (5) lack of knowledge about the opportunity to develop and discuss a work reintegration plan before surgery with the employer and an occupational physician. In addition, important environmental conditions of patients’ behavior are considered to be: (1) inconsistency and lack of clarity in convalescence recommendations given by gynecologists, family physicians, and occupational physicians; (2) lack of communication among gynecologists, family physicians, and occupational physicians; (3) lack of clarity from health care providers about who to contact in case of postoperative complaints; (4) lack of initiative of the employer and/or occupational physician to develop and discuss a work-reintegration plan before surgery with the employee; and (5) lack of involvement of the employer and occupational physician during the perioperative and reintegration period.

### Step 2: Matrices

In total, 12 performance objectives derived from the main behavior objective were formulated (see Table 1).

**Table 1:** Performance objectives to empower gynecological patients during the perioperative period and return to normal activities and work to obtain timely RTW and prevent work disability.

Who	Performance objectives
Patients	Acquaint themselves with important information including: realistic detailed convalescence recommendations regarding RTW activities; the importance of timely and gradual resumption of work activities after the surgical procedure; the technical aspects of surgical procedures; the admission process at the hospital; the kind of anesthesia that will be used during surgery; main complications that could happen during and after surgery; symptoms that can be expected after surgery (eg, vaginal blood loss and intestinal complaints); the cosmetic consequences of surgery; main psychological consequences of hysterectomy or adnexal surgery; telephone numbers of experts (eg, gynecologist, social workers, and homecare services); what to do and who to contact in case of physical or mental postoperative complaints or delayed recovery; and the risks of work disability after surgery.
	Do not extent their sick leave period beyond recommended period on own initiative.
	Develop a work-reintegration plan.
	Discuss their personalized work-reintegration plan with their employer and/or occupational physician.
	Identify possible barriers for a safe and appropriate RTW.
	Exchange experiences with other patients who underwent the same surgery.
	Receive answers to individual questions and uncertainties about recovery and RTW.
Gynecologists and family physicians	Acquaint themselves with uniform, detailed convalescence recommendations for their patients.
Occupational physicians	Acquaint themselves with detailed convalescence recommendations for their patients.
	Provide the opportunity to develop a work-reintegration plan before surgery.
Employers	Provide the opportunity to develop a work-reintegration plan before surgery.
	Discuss the personalized work-reintegration plan composed by their employees.
	Show involvement with their employee during the perioperative and reintegration period.

In addition to performance objectives for patients, there were also performance objectives formulated for gynecologists, family physicians, occupational physicians, and employers (Table 1). Nevertheless, the primary focus during the next steps of the IM protocol (the development of the eHealth intervention) will be on patients. Ideally, for each group (patients, gynecologists, family physicians, occupational physicians, and employers) an intervention should be developed specific to their needs, wishes, and behavior outcomes to minimize the risks of theory and/or program failure. However, a balance had to be found between the ideal situation and what was within reach of this study. Secondly, the performance objectives of gynecologists, family physicians, occupational physicians, and employers could be considered external determinants of patients’ behavior. These determinants can either be influenced by the patients or the patients can learn these skills through the intervention and how to handle them adequately. Finally, the performance objectives of gynecologists, family physicians, occupational physicians, and employers are relatively simple objectives to reach. The researchers are convinced that the main part of these objectives can be reached through making agreements with gynecologists, family physicians, occupational physicians, and employers and by involving them in the evaluation and implementation plan (IM Steps 4-6) without specifying determinants of their behavior and applying specific theoretical methods and strategies for them.

The literature search showed that the main determinants of recovery and return to normal activities and work (in addition to the physical condition of the patient, level of invasiveness of the surgical procedures, and related complications) are the patients’ attitude, social influence, and self-efficacy [60-65]. In addition, skills, barriers, and facilitators are important factors that influence RTW [66-68]. For these reasons, the Attitude-Social influence-self-Efficacy (ASE) model [69,70], adapted for recovery and return to normal and work activities, was used to affect behavior of patients (see Appendix 1). The ASE model is comparable to the theory of planned behavior [71], which describes the relation between attitude and behavior. The modified ASE model describes that the behavior of a patient after surgery regarding recovery and return to normal and work activities is determined by attitudinal beliefs, social influence, and self-efficacy beliefs, and is influenced by skills, barriers, and resources. The ASE model was used to create matrices with change objectives. To fill out the matrices, available literature regarding the performance objectives and determinants was studied together with the results of the needs assessment and expertise of the project group. Appendix 2 presents an example of the change objective “Patients develop a work-reintegration plan.”

### Step 3: Theory-based Methods and Practical Strategies

Numerous practical methods and suitable strategies to affect all formulated determinants were identified and used for the development of tools and materials of the eHealth intervention. Appendix 3 presents some examples of these methods with preconditions for the method necessary for it to succeed [72] and final tool/materials of the eHealth intervention. References and footnotes explain the source and development process of each method, strategy, and tools/materials.

### Step 4: Program Plan/Design of the Intervention

With the knowledge obtained in the previous steps of the IM protocol, the project group convened at several meetings to invent various appropriate tools for the eHealth intervention. A website producer specializing in eHealth interventions and a screenwriter were consulted at some of the meetings. In addition, an experienced gynecologist outside the project team was consulted to judge the medical content of one of the tools.

In close collaboration with the website producer, the eHealth intervention was developed with MODX, an open-source hypertext preprocessor (PHP) Web application framework with a capable built-in content management system (CMS). The Internet address of the eHealth intervention is “http://www.ikherstel.nl/www.ikherstel.nl,” which means, “I am recovering” (Appendices 6-10) [73]. The eHealth intervention was developed with special attention to colors, layout, navigation, and readability to create confidence and user-friendliness. For the patient, it consists of two main sections: an Action List to assist in resumption of activities and a central home page. Gynecologists, family physicians, and occupational physicians have access to a different section. Table 2 presents an overview of the tools in the eHealth intervention. For some tools, additional information about the development and functioning is described subsequently.

**Table 2:** Structure of the eHealth intervention.

Tool		Content	Target
**Action list**			
	Compose a work-reintegration plan	Tool to compose a detailed reintegration plan with adaptations for work if necessary.	Patient, employer, occupational physician
	Resume normal activities	Tool to compose detailed advice about when normal (private) activities can be carried out again	Patient, family
	Evaluate complications	Estimate severity and consequences of a complication	Patient, gynecologist
	Recovery monitor	Monitoring recovery and offering assistance when relevant	Patient
	Satisfaction with recommendations	Evaluation and explanation of convalescence recommendations	Patient
	Satisfaction with the recovery process	Evaluation of satisfaction with recovery and reintegration process. Provision of advice regarding which care provider(s) to approach to receive appropriate help, when relevant.	Patient
	Invite employer	Invite employer for (anonymous) section of the eHealth intervention which includes video and recommendations	Patient, employer
**Home page**			
	Video	Illustrate common pitfalls during the perioperative and reintegration period	Patient, employer, gynecologist
	Recommendations for employee	Advice for a successful reintegration	Patient
	Recommendations for employer	Advice for appropriate involvement regarding employee during the perioperative and reintegration period	Employer
	Frequently asked questions	Extensive list of answers and pictures to most frequently asked questions	Patient
	Glossary	Explanation of most frequently used medical terms	Patient
	Forum	Ability to interact in public or through private messages with other patients	Patient
	Links to other websites	Relevant websites concerning the perioperative and reintegration period	Patient
**Section aimed at gynecologists, family physicians, and occupational physicians**			
	Guidelines	Well-defined convalescence recommendations after hysterectomy and laparoscopic adnexal surgery	Gynecologists, family physicians, occupational physicians
	Casuistry	Indications, perioperative course, and recovery regarding hysterectomy or laparoscopic adnexal surgery	Gynecologists, family physicians, occupational physicians
	Background information	Specialistic information regarding different kinds of hysterectomy and laparoscopic adnexal surgery	Gynecologists, family physicians, occupational physicians

#### Action List

When a patient logs onto the eHealth intervention, she will be immediately directed to the Action List. This list consists of different tools developed to target specific determinants, aimed at encouraging return to work activities, coaching patients in case of uncertainties, answering possible questions, prevention of common pitfalls, and improving communication among the patient, care providers, and the employer. An algorithm based on the date of surgery determines the priority in which the different actions should be performed to improve the recovery process. Tools of the action list are:

##### Composition of a Work-Reintegration Plan

By using this tool, the patient is able to select activities that are required to fulfill her work activities and at what level (eg, lifting 5 kilograms or walking 1 hour.). Consequently, on the basis of the operation date and how the surgery went (input of gynecologist), the eHealth intervention provides the patient with tailored advice about when these activities are thought to be medically safe to resume. The recommendations are based on the results of a modified Delphi study, in which an expert panel of gynecologists, family physicians, and occupational physicians developed detailed multidisciplinary convalescence recommendations for resumption of work activities after hysterectomy and/or laparoscopic adnexal surgery [74]. Moreover, this part of the eHealth intervention provides an overview of potential bottlenecks for reintegration and motivates patients to consider if work adaptations are required temporarily. A printout can be made to discuss the advice with the employer and/or occupational physician to develop an extended reintegration plan.

##### Resumption of Normal Activities

This functionality guides the patient to compose a detailed tailored plan about the gradual resumption of various daily activities (eg, climbing stairs and vacuum cleaning). Recommendations are based on the results of the modified Delphi study [74]. This tool also evaluates if help is needed for tasks such as housekeeping or taking care of young children. A printout can be made to share with relatives or friends.

##### Evaluation of Complications

When a complication has occurred, the eHealth intervention carefully determines through a survey which symptoms require additional consultation with care providers or adaptation of the convalescence recommendations. The project group developed the survey and determined which symptoms are severe complications. If the tool is not able to provide recommendations under these circumstances, an email will be sent to inform the gynecologist of the condition of the patient in order to evaluate her symptoms and possible consequences.

#### Home Page

##### Video

Because of the influence of modeling behavior on attitude, a video was chosen as the most appropriate medium to deliver an informative message to patients and relevant stakeholders about common pitfalls during the perioperative and reintegration period. The video aims to prevent these problems by stimulating patients and employers to discuss potential problems and to develop a reintegration plan to facilitate and improve reintegration. The experiences of the patients in the focus group discussions were converted into common pitfalls for patients, employers, and health care providers during this period, and a screenwriter processed them into a script for a video showing two cases of a good and bad interaction between a patient and her environment. The screenwriter worked together closely with 3 gynecological patients to make the video geared to the patients’ perception of the perioperative and reintegration period.

##### Recommendations for Employee and Employer

Based on the experiences of the patients in the focus groups, the researchers formulated main recommendations for patients and employers regarding a successful reintegration.

##### Frequently Asked Questions

Answers to questions brought up during the focus group discussions and those found as main topics in patients’ brochures or in discussions of gynecological patients on the Internet were formulated by the researchers (based on the literature and clinical experience) and put into patient leaflets. An experienced gynecologist outside the project team judged all questions and answers on reliability and clarity, and suggested possible adjustments.

##### Glossary

Based on the literature, an explanation of the most frequently used medical terms was provided by the researchers.

##### Links to Other Websites

The researchers searched the Internet for the most relevant websites for gynecological patients and made a selection based on relevance, reliability, and clearness of the information.

#### Section Aimed at Gynecologists, Family Physicians, and Occupational Physicians

##### Guidelines

Multidisciplinary guidelines with well-defined convalescence recommendations after uncomplicated hysterectomy (laparoscopic supracervical, total laparoscopic/laparoscopic-assisted, vaginal, and abdominal) and laparoscopic adnexal surgery on benign indication are provided. Recommendations are based on a modified Delphi study.

##### Casuistry

Classic examples of indications for surgery, perioperative course, and recovery after uncomplicated hysterectomy or laparoscopic surgery were developed based on literature and clinical experience of the project group.

##### Background Information

Elucidation of different types of hysterectomy and laparoscopic adnexal surgery concerning surgical technique, level of invasiveness, and medical consequences were formulated by the researchers.

Test Phase

Fifteen patients, 11 physicians (gynecologists, family physicians, and occupational physicians), 3 eHealth specialists, and 1 representative of a patient organization completed the evaluation form regarding the demo version of the eHealth intervention. Appearance and behavior prescriptions were judged by most as pleasant, conveniently arranged, and helpful. With regard to burdens of using the eHealth intervention, almost all respondents judged the application navigation as clear and the intervention length as appropriate. However, a manual providing an overview of the different tools of the eHealth intervention was found desirable by only one of the respondents. Furthermore, two software incompatibility problems were reported. Concerning the content of the information, the way it was delivered, and the message (source and style), most of the respondents were satisfied and expected that it could empower patients, employers, and physicians. Remarks for improvement were related to supplying more detailed information about the surgery, possible psychological complications after the operation, less complicated sentences, and a more prominent place for the source of the information. Finally, participation of the patient in the treatment and the eHealth intervention’s ability to assess and tailor the recommendations to empower patients during the perioperative period and return to work activities were judged as helpful by most of the respondents. There were no suggestions for improvement of these features.

The patients indicated that their input provided during the focus group discussions was recognizably integrated into the intervention. Additionally, almost all patients confirmed that they would recommend the eHealth intervention in the current form to a friend.

#### Modifications Based on the Test Phase

As described previously, the respondents did not request major revisions of the eHealth intervention and only minor adjustments were proposed. Therefore, none of the original developed tools were removed from the eHealth intervention and no new functionalities were added. Following up on the suggested improvements, a manual with directions for use was added to the eHealth intervention, incompatibility problems with different kinds of software were solved, some information on the eHealth intervention was elaborated on and explained in simplified sentences, and the logo of the university hospital was added in a prominent place on the eHealth intervention. This resulted in the final eHealth intervention that was used to perform a randomized controlled trial (RCT) [75]. Screenshots of the eHealth intervention can be found in Appendices 6-10.

### Step 5: Design of an Implementation Plan

In this study, anticipation of adoption and implementation started with the involvement of patients (target group) in all stages of the intervention development and evaluation. Health care providers, occupational physicians, and eHealth specialists participated in the evaluation of the intervention during IM Step 4. In addition, a committee with representatives of the Dutch medical boards of gynecologists, occupational physicians, and family physicians, and a representative of an umbrella patient organization were involved during the development of all steps of the intervention and agreed to stay involved during the final implementation steps of this intervention. Through this committee, a linkage system was created by involving the future users and implementers of the intervention from the start of the intervention development process. Furthermore, an important target of this study was to develop an eHealth intervention that could be used by patients, doctors, and employers without any support to simplify implementation. Evaluation of self-reliant use by patients and important stakeholders was evaluated positively during the test phase of Step 4.

Within the context of a RCT with the eHealth intervention (Step 6), the project group will facilitate its implementation and maintenance. In collaboration with the relevant care providers, the eHealth intervention will be offered as a supplement to standard perioperative care and will involve minimal additional time investment for the care providers. Agreements about usage of the contents of the eHealth intervention will be made with the gynecologists of participating hospitals and the family physicians and occupational physicians of participating patients. Therefore, the main purpose of this step was to create familiarity and support for the eHealth intervention and convalescence recommendations by all prospectively involved users. To reach these purposes for all of the different user groups, information letters will be distributed among patients and care providers. In addition, presentations with background information about the development of the eHealth intervention, its contents, and how to use it will be given to the gynecologists during general teaching meetings at their hospitals. Employers will become familiar with the intervention through invitation for participation by the patients (ie, employees). The eHealth intervention will be primarily used during the period of sick leave after surgery. Therefore, no agreement with the patients’ employers to use the eHealth intervention during work hours will be made.

With the information gathered during the process evaluation (Step 6), in collaboration with the committee with representatives of the Dutch medical boards of gynecologists, occupational physicians, and family physicians, and the patient organization, a final implementation plan will be developed. In this plan, medical insurance companies and the Health Care Insurance Board (CVZ) will likely be involved for the final implementation of the eHealth intervention.

### Step 6: Evaluation Plan

The evaluation of the eHealth intervention will be performed by a RCT, during which the eHealth intervention will be compared with usual given care at 7 participating medical centers [75]. A power calculation was performed on the primary outcome (sustainable RTW) and showed that a total participation of at least 212 patients, their health care providers, and employers should be the goal. Patients will be recruited to participate in the RCT when they are placed on a waiting list for a hysterectomy or laparoscopic adnexal surgery on benign indication in one of the 7 participating medical centers, are aged 18-65, and they work (either paid or unpaid) for at least 8 hours per week. The main exclusion criteria are malignancy, deep infiltrating endometriosis, concomitant surgical procedures, major comorbidity, sick-listed for more than 2 months, currently in a lawsuit against their employer, and not able to use the Internet or unable to understand the Dutch questionnaires. If a patient participates, the researchers will inform her family physician and occupational physician by letter about the content of the intervention, the group allocation, and what is expected of them regarding the provision of health care. Follow-up will take place approximately 26 weeks after surgery.

Patients willing to participate and who meet the inclusion criteria will be randomized to the intervention or usual care group (control group). Main outcome measures of the RCT are the effectiveness of the eHealth intervention compared to usual care with respect to RTW, general recovery, quality of life, pain intensity, and complications. Part of the RCT will be a process evaluation of the patients, their care providers, and employers in the intervention group. Main outcome measures of the process evaluation are the extent to which the eHealth intervention and convalescence recommendations are used and followed up (compliance); appreciation of the different tools of the eHealth intervention and advice; perceived effectiveness, usage, and implementation barriers; and suggestions for improvement.

The outcome measures will be obtained using questionnaires administered at baseline and at 2, 6, 12, and 26 weeks after surgery. Gynecologists will complete questionnaires 1 day after surgery for each patient and at the end of the study. Employers will be asked to evaluate the eHealth intervention 8 weeks after their employee’s surgery.

The study design and procedures of the RCT study were approved by the Medical Ethics Committee of the VU University Medical Center (#2009/218, October 22, 2009).

## Discussion

### Main Findings

In this study, the IM protocol turned out to be a useful method to develop and tailor an eHealth intervention aimed at the empowerment of gynecological patients during the perioperative period including return to normal activities and work. By using available literature and focus group discussions, it became increasingly clear that to obtain timely RTW and prevent work disability, the intervention should target both behaviors of patients as well as environmental determinants. Performance objectives for obtaining timely RTW and prevention of work disability were formulated and matrices with change objectives, explaining how patients and their environment have to change as a result of the eHealth intervention to reach the performance objectives, were developed. Finally, based on the ASE model [69,70], theoretical methods and practical strategies, suitable tools, and materials for the eHealth intervention were developed. Most of the participating patients and stakeholders judged the intervention to be a promising eHealth tool to empower gynecological patients during the perioperative period to return to their normal activities, including work.

### Strengths and Limitations

A primary strength of this study lies in the way the eHealth intervention was developed, tailored, and assessed. Both theory and evidence were combined and patients and most relevant stakeholders were involved, minimizing the risks of theory and/or program failure [72]. The frequent involvement of patients in several steps of the IM process resulted in an eHealth intervention that was specifically tailored to their needs and wishes and therefore more likely to be implemented successfully*. *In addition to information supply, which is the primary aim of most websites, this eHealth intervention distinguishes itself by monitoring the recovery process, giving tailor-made advice based on patients’ workloads, and informing patients when additional consultation of care providers is needed. By linking patients with their gynecologists, convalescence recommendations can be adapted and insecurities regarding consequences of the complications can be solved. Connecting patients and employers facilitates a dialogue and the joint effort to compose a reintegration plan. Furthermore, this eHealth intervention is developed to be used without support and with minimal effort of care providers. Therefore, use of the intervention costs little and implementation is expected to be relatively easy. Moreover, like most eHealth interventions, an important strength is the possibility to use it at the time, place, and pace that fits the patient, care provider, and employer [38]. Finally, the combined approach of encouraging and helping patients to participate in their consultation and empowering clinicians with skills to identify and adapt to the needs of their patients is thought to produce long-term benefits for patients [21].

Main limitations concerning the needs assessment of this study include a possible selection bias; patients assigned to the focus group discussions are a selection of the patients willing to discuss their perioperative problems. Patients less willing to discuss their problems may also experience different perioperative issues. However, through purposeful sampling and by proactively approaching all relevant patients for participation in the focus group discussions, we tried to minimize this selection bias as much as possible. In addition, the influence of dominant patients who might be overly influential cannot be excluded. On the other hand, specific observations on this matter showed that this rarely occurred [Pittens et al, unpublished data, 2012]. Furthermore, these patients already underwent the surgery, whereas the intervention is designed to be used both before and after surgery. It has to be determined whether this intervention is applicable to the entire target population and whether the intervention fits the needs of patients both before and after surgery. Due to practical reasons, not all stakeholders (eg, employers and health care providers) were involved in the needs assessment and development process of this eHealth intervention. As a consequence, the intervention might be less supported by these groups. However, results of prior focus group discussions with supervisors and care providers in another comparable IM study [47] were used and some of those stakeholders were also involved in the test phase. Because this was an exclusively Dutch study directed at the Dutch health care system, a final limitation is that external validity of the eHealth intervention has to be examined before the results may be applied internationally.

### Comparison with Other Studies

To our knowledge, this is the first study that tailors an eHealth intervention through the IM protocol to empower gynecological patients during the perioperative period to obtain timely RTW and prevent work disability. Therefore, comparison with other studies is limited. However, previous research showed several developmental and interventional characteristics. For example, it was demonstrated that IM is a successful method to tailor eHealth [45,76] as well as RTW [47,48] interventions. Moreover, Web-based interventions show positive effects on empowerment [25]. Furthermore, it is proven that tailoring an eHealth intervention influences usage positively (eg, time and frequency) and increases the effectiveness of the message [77,78]. In contrast to most eHealth interventions, this intervention aims at secondary and tertiary prevention. Therefore, further research is needed to determine whether the characteristics mentioned previously also apply to the present study.

Although comparable studies are lacking, the approach followed in this study—involving relevant stakeholders in the development of an eHealth intervention—is in line with an observed trend of multi-stakeholder involvement in health care in general [79,80]. Gained experiences in this study might contribute to additional insights for future initiatives on multi-stakeholder involvement in health care.

### Interpretation of the Results and Policy Implications

This study shows that the IM protocol can successfully be used for the development and tailoring of an eHealth intervention for gynecological patients. The protocol led to a systematic development of the intervention, it made sure that collaboration with the main target group was realized, and both theory and evidence was used to tailor the intervention.

Furthermore, through the detailed convalescence recommendations provided by the eHealth intervention, patients will be better informed about when it is thought to be medically safe to resume daily and work activities after gynecological surgery and it will give them the possibility to arrange workplace adaptations if necessary [74]. Prospective cohort studies exploring sick leave after general surgical procedures show that return to work is primarily influenced by the expectations of the patient and their supervisors rather than physical factors or the type of surgery [1,10,81]. Therefore, it is assumed that these tailor-made convalescence recommendations will help to accelerate recovery and stimulate patients to resume activities with increasing gradations of strain, which will presumably bring about a quicker recovery and RTW and prevent work disability [82-84]. Therefore, it is expected that this eHealth intervention fulfills patients’ needs and is able to empower gynecological patients during the perioperative period and return to normal activities and work [77]. However, its adoption, barriers for usage in daily practice, and implementation possibilities by patients and stakeholders still need to be evaluated more extensively in a process evaluation. Furthermore, a RCT will be needed to assess the effect of empowering gynecological patients during the perioperative period and return to normal activities and work by this eHealth intervention on work disability prevention, resumption of activities, and quality of life [75]. The results are important to assess this intervention’s true value and policy implications.

This eHealth intervention is developed for patients who underwent a hysterectomy or laparoscopic adnexal surgery. However, the strategy used to develop the intervention and the final result may also be used as a blueprint for other kinds of surgical procedures.

### Conclusion

The development of an eHealth intervention according to the IM protocol to obtain timely RTW and prevent work disability by empowerment and improving communication after gynecological surgery resulted in an intervention based on both theory and evidence and involvement of patients and most stakeholders. This eHealth intervention is well accepted by patients and stakeholders and is considered to be a promising tool to obtain timely RTW and prevent work disability after gynecological surgery. Its effectiveness needs to be proven in a RCT [75].

## Multimedia Appendix 1

Attitude Social influence-self-Efficacy model (ASE) [69,70] adapted for recovery and Return to Normal Activities (RNA) and Return to Work (RTW) after gynaecological surgery.

## Multimedia Appendix 2

Example of change objectives of patients.

## Multimedia Appendix 3

Theoretical methods and practical strategies for recovery and return to normal and work activities.

## Multimedia Appendix 4

Evaluation questionaire of the eHealth intervention ikherstel.nl for focus group participants.

## Multimedia Appendix 5

Evaluation questionaire of the eHealth intervention ikherstel.nl for professionals.

## Multimedia Appendix 6

Screenshot of http://www.ikherstel.nl.

## Multimedia Appendix 7

Screenshot of http://www.ikherstel.nl.

## Multimedia Appendix 8

Screenshot of http://www.ikherstel.nl.

## Multimedia Appendix 9

Screenshot of http://www.ikherstel.nl.

## Multimedia Appendix 10

Screenshot of http://www.ikherstel.nl.

## Abbreviations

ASE: Attitude–Social influence–self-Efficacy
CMS: content management system
IM: intervention mapping
RCT: randomized controlled trial
RTW: return to work
