# Supplementary material for: eHealth Program to Empower Patients in Returning to Normal Activities and Work After Gynecological Surgery: Intervention Mapping as a Useful Method for Development
Source: J Med Internet Res. 2012 Oct 19;14(5):e124. doi: 10.2196/jmir.1915 (PMC3510728; doi:10.2196/jmir.1915)
Supplement: Supplementary file 1 [file jmir_v14i5e124_app1.pdf]

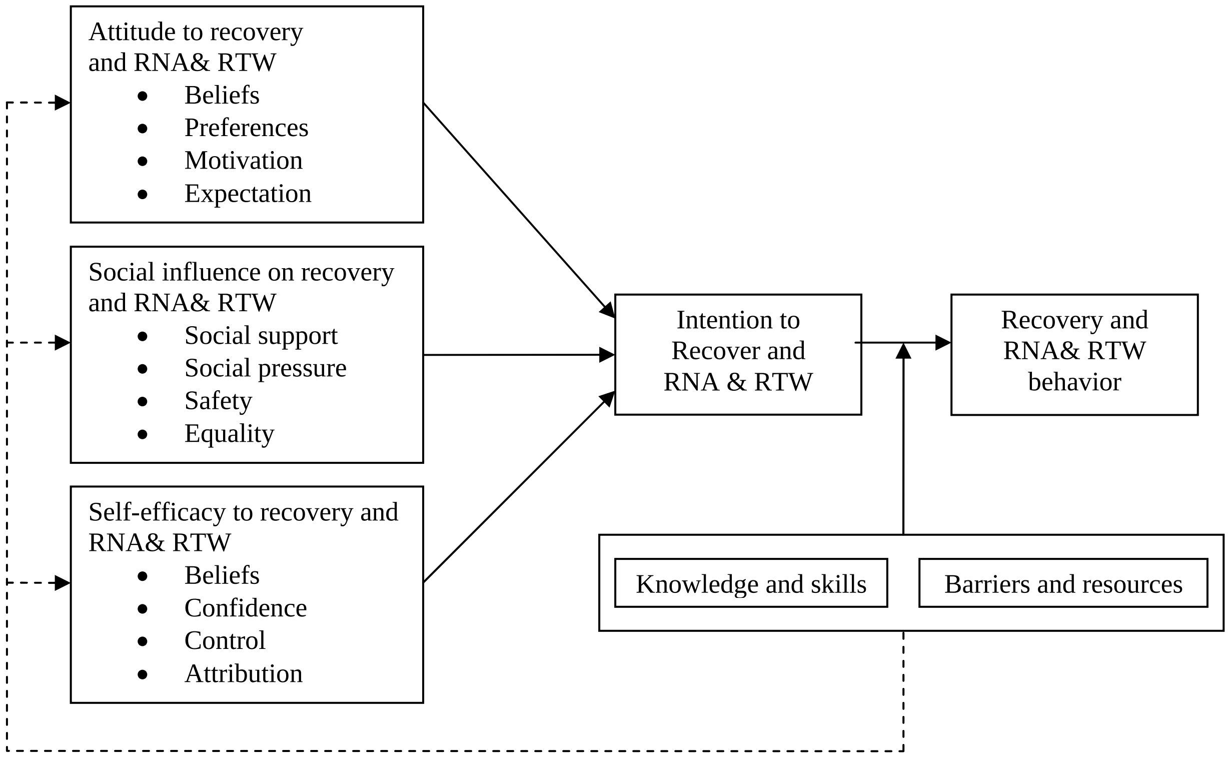

Attitude Social influence-self-Efficacy model (ASE) [69, 70] adapted for recovery and Return to Normal Activities (RNA) and Return To Work (RTW) after gynecological surgery
