# Supplementary material for: eHealth Program to Empower Patients in Returning to Normal Activities and Work After Gynecological Surgery: Intervention Mapping as a Useful Method for Development
Source: J Med Internet Res. 2012 Oct 19;14(5):e124. doi: 10.2196/jmir.1915 (PMC3510728; doi:10.2196/jmir.1915)
Supplement: Supplementary file 2 [file jmir_v14i5e124_app2.pdf]

Performance objective	Personal determinants				External determinants	
	Attitudinal beliefs	Knowledge	Skills	Self-efficacy beliefs	Barriers	Support
**Patients develop a work-reintegration plan**	• Are willing to accept the convalescence recommendations [a][2]<br>• Acknowledge that RTW in an early stage is important for their health [a][8]<br>• Take the effort to develop a reintegration plan[b]	• Know about the importance of gradual resumption of activities after gynecological surgery[c]<br>• Know about health and financial consequences of work disability [a, b, d]<br>• Know about the risks of work disability after surgery [a][8]	• Have skills to make a reintegration plan[b]<br>• Find solutions to identify possible barriers for RTW [a][1]	• Are confident concerning the tailor made medical content of their RTW-plan[b]<br>• Feel self confident about discussing the RTW-plan with their employer[b]	• Feel encouraged by Health care providers to RTW [a][1,82]<br>• Employers give the opportunity to formulate a reintegration plan before surgery[b]	• Receive help (if necessary) of their OPs by composing a reintegration plan [a][59]<br>• Feel receptiveness of their employers and OPs to think about adjustments in order to make an appropriate reintegration plan [a][67]<br>• Different expectations regarding RTW are prevented by discussion of work-reintegration plan with employers [a][1]

[a] [..] See reference list
[b] Information obtained in the focus group discussions
[c] Expertise of the project group
[d] Cassidy JD, Carroll LJ, Cote P, Lemstra M, Berglund A, Nygren A. Effect of eliminating compensation for pain and suffering on the outcome of insurance claims for whiplash injury. N Engl J Med 2000 April 20;342(16):1179-1186. PMID:10770984.
