# Supplementary material for: eHealth Program to Empower Patients in Returning to Normal Activities and Work After Gynecological Surgery: Intervention Mapping as a Useful Method for Development
Source: J Med Internet Res. 2012 Oct 19;14(5):e124. doi: 10.2196/jmir.1915 (PMC3510728; doi:10.2196/jmir.1915)
Supplement: Supplementary file 3 [file jmir_v14i5e124_app3.pdf]

Determinant	Methods	Precondition	Strategy: Tools/Materials	Tool of eHealth intervention *(table 2)*
**Attitudinal beliefs**	• Persuasive Communication [d]	▪ Credibility and clarity of the source<br>▪ Knowledge of Gynecologists, OPs and GPs about multidisciplinary guidelines	➢ Uniform advice of Gynecologists, OPs and GPs through implementation of a multidisciplinary guideline with well-defined convalescence recommendations after all types of hysterectomy and laparoscopic adnexal surgery. In the eHealth intervention, the care providers can find the guidelines, information about different types of surgery and casuistry.[a][58,59,74], [b]	✓ Guidelines<br>✓ Casuistry<br>✓ Background information
			➢ Provide reasons why prolonged recovery time and sick leave is not desirable can be found on the eHealth intervention.[a][8], [b]	✓ Frequently asked questions
			➢ In order to have a reliable layout, the eHealth intervention is developed with attention for colours and correct comprehensible usage.[b, c]	
	• Self re-evaluation [a][42]	▪ Motivation	➢ Recovery monitoring by the eHealth intervention and feedback in case of abnormal or unrealistic recovery beliefs or RTW time.[a][56], [c]	✓ Recovery Monitor
	• Modelling [a][42]	▪ Acceptance and reinforcement of the model	➢ The eHealth intervention provides a video with models expressing tools for an appropriate recovery and RTW.[b, c]	✓ Video
	• Goal setting [a][42]	▪ Acceptance of recommendations<br>▪ Right balance between feasibility and challenge	➢ The eHealth intervention encourages and provides tools to compose a reintegration plan, including a schedule for private life and the home situation.[a][59], [b]	✓ Compose reintegration plan<br>✓ Resume activities
			➢ Possibility to print out the individualized reintegration plan to discuss it with the employer and/or OP.[b, c]	✓ Compose reintegration plan<br>✓ Resume activities
			➢ The eHealth intervention provides realistic information regarding the recovery and RTW process.	✓ Recommendations for employee<br>✓ Frequently asked questions
**Social influence**	• Mobilizing social support from family and friends [a][42]	▪ Acceptance of assistance<br>▪ Helpful family and friends	➢ The eHealth intervention encourages and provides tools to consider the need for social support during the recovery period.[b, c]	✓ Resume activities<br>✓ Frequently asked questions
	• Mobilizing support from other patients [a][42]	▪ Openness about surgery and recovery to other patients	➢ The eHealth intervention contains a forum to contact other patients (peer support) and has functionality to send private messages. With these tools, personal strategies to deal with difficult situations and barriers may be shared and meetings can be organized if needed.[b]	✓ Forum<br>✓ Links to other websites
	• Mobilizing social support / create openness and respect from work environment [a][42]	▪ Openness about (implications of) surgery to employer<br>▪ Involvement of employer	➢ The employee can invite her employer for a section of the eHealth intervention, which shows common pitfalls and provides tools to help support the patient during the peri-operative period and RTW process.[c]<br>➢ Possibility to print out the reintegration plan and share it with the employer.[b,c]	✓ Video<br>✓ Recommendations employee<br>✓ Recommendations Employer
	• Create openness with partner [a] [42]	▪ Comprehension of partner	➢ Possibility to print out individualized convalescence recommendations to share with partner.[b]	✓ Resume normal activities

[a] [..] See reference list
[b] Information obtained in the focus group discussions
[c] Expertise of the project group
[d] Strader MK, Katz BM. Effects of a persuasive communication on beliefs, attitudes, and career choice. J Soc Psychol 1990 April;130(2):141-150. PM:2342343.
