# Supplementary material for: eHealth Program to Empower Patients in Returning to Normal Activities and Work After Gynecological Surgery: Intervention Mapping as a Useful Method for Development
Source: J Med Internet Res. 2012 Oct 19;14(5):e124. doi: 10.2196/jmir.1915 (PMC3510728; doi:10.2196/jmir.1915)
Supplement: Supplementary file 4 [file jmir_v14i5e124_app4.pdf]

Evaluation questionaire of the eHealth intervention 'ikherstel.nl'

Questions asked to the focus group participants

1. Please indicate the degree you agree or disagree with the statement. In the last column you are able to explain your answer.

	Completely disagree	Disagree	Neutral	Agree	Completely agree	Explanation
The website was easy accessible	○	○	○	○	○	
It was clear to me where I had to log into the website	○	○	○	○	○	
Navigating the website was easy	○	○	○	○	○	
Finding answers to personal questions was easy	○	○	○	○	○	
Obtaining recommendations for resumption of activities was easy	○	○	○	○	○	
It was clear to me where I could get help for problems regarding the website	○	○	○	○	○	
I appreciated the layout of the website	○	○	○	○	○	
The website is conveniently arranged	○	○	○	○	○	
The texts on the website were easy to understand	○	○	○	○	○	
The website is a good support for patients who want to resume their daily activities	○	○	○	○	○	
The website is a good support for patients to communicate with their employer about reintegration	○	○	○	○	○	
The website offers enough opportunities to exchange experiences with other patients	○	○	○	○	○	

2. Please indicate on a five point scale how helpful the following tools of the website are:

	Not helpful at all	Not helpful	Neutral	Helpful	Very helpful	Not applicable
The movie on the website is	○	○	○	○	○	○
The links to other websites are	○	○	○	○	○	○
The forum on the website is	○	○	○	○	○	○
The FAQ−list is	○	○	○	○	○	○

3. Are there unnecessary tools on the website?

○ Yes, namely: ______

○ No
