# Supplementary material for: eHealth Program to Empower Patients in Returning to Normal Activities and Work After Gynecological Surgery: Intervention Mapping as a Useful Method for Development
Source: J Med Internet Res. 2012 Oct 19;14(5):e124. doi: 10.2196/jmir.1915 (PMC3510728; doi:10.2196/jmir.1915)
Supplement: Supplementary file 5 [file jmir_v14i5e124_app5.pdf]

Evaluation questionaire of the eHealth intervention 'ikherstel.nl'

Questions asked to medical doctors, eHealth specialists and a respresentative of a patient organisation

1. Please give your opinion about the different tools of the eHealth intervention ikherstel.nl (e.g. action list, movie, recommendations for employee/employer, FAQs, forum, guidelines, etc)?

2. Was it easy to navigate the website?

3. Are the texts on the website easy to understand?

4. Are there tools on the website that did not work properly?

5. Do you have any ideas to improve this website?

6. Do you have any other comments?
