# Supplementary material for: eHealth Program to Empower Patients in Returning to Normal Activities and Work After Gynecological Surgery: Intervention Mapping as a Useful Method for Development
Source: J Med Internet Res. 2012 Oct 19;14(5):e124. doi: 10.2196/jmir.1915 (PMC3510728; doi:10.2196/jmir.1915)
Supplement: Supplementary file 6 [file jmir_v14i5e124_app6.pdf]

startpagina | contact | colofon |

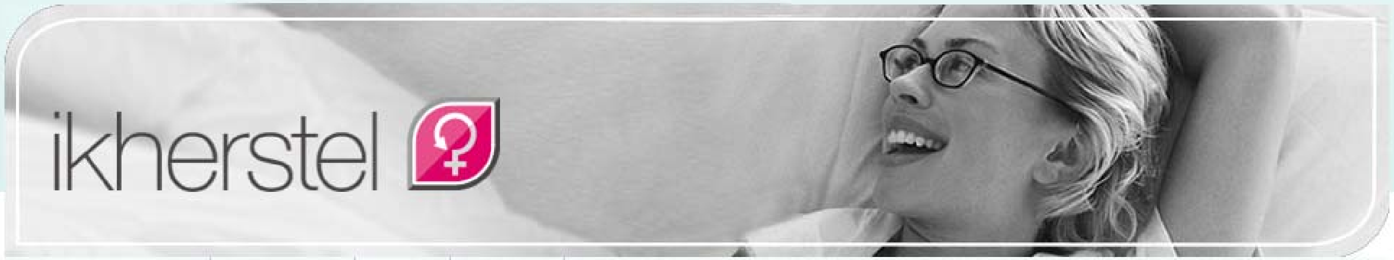

het onderzoek | deelname | team | nieuws

Welkom bij ikherstel.nl

Deze website heeft tot doel om ondersteuning te bieden rondom (een aantal soorten) gynaecologische operaties. Momenteel worden verschillende vormen van zorg in onderzoeksverband geëvalueerd.

Wanneer u aan dit onderzoek deelneemt, kunt u rechts van deze tekst inloggen met uw gebruikersnaam en wachtwoord.

Wilt u deelnemen aan het onderzoek? Bekijk de informatie voor deelname

Inloggen als deelnemer

gebruikersnaam:

wachtwoord:

Klik hier als u uw wachtwoord bent vergeten

Inloggen

VU medisch centrum

ZonMw

Copyright © 2009: ikherstel.nl

Copyright © 2009: ikherstel.nl
