# Supplementary material for: eHealth Program to Empower Patients in Returning to Normal Activities and Work After Gynecological Surgery: Intervention Mapping as a Useful Method for Development
Source: J Med Internet Res. 2012 Oct 19;14(5):e124. doi: 10.2196/jmir.1915 (PMC3510728; doi:10.2196/jmir.1915)
Supplement: Supplementary file 7 [file jmir_v14i5e124_app7.pdf]

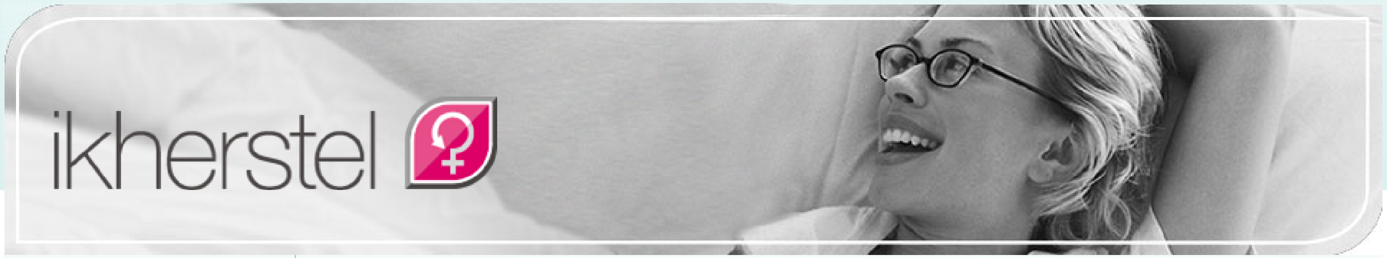

Actielijst

Hieronder, in het roze, staan de onderdelen welke van belang zijn om op korte termijn uit te voeren. Wanneer u een onderdeel van deze actielijst heeft afgerond, schuift het betreffende roze blok naar rechts en krijgt een grijze kleur.

- tevredenheid adviezen en herstel
- complicaties
- herstelmonitor
- vragenlijsten

Overige Items

Hieronder, in het grijs, staan de onderdelen waarmee u voor het onderzoek niet op korte termijn iets hoeft te doen maar die wel beschikbaar zijn voor gebruik.
De blauw gekleurde functionaliteiten adviseren we om zowel voor als na de operatie regelmatig te raadplegen.

- advies hervatten werkactiviteiten
- advies hervatten privé activiteiten
- uitnodigen leidinggevende

Snelmenu

- Mijn gegevens
- PDF privé advies
- PDF werk advies
- Nodig uw leidinggevende uit om de website te bekijken
- Handleiding bij gebruik website

Copyright © 2009: ikherstel.nl
