# Supplementary material for: eHealth Program to Empower Patients in Returning to Normal Activities and Work After Gynecological Surgery: Intervention Mapping as a Useful Method for Development
Source: J Med Internet Res. 2012 Oct 19;14(5):e124. doi: 10.2196/jmir.1915 (PMC3510728; doi:10.2196/jmir.1915)
Supplement: Supplementary file 8 [file jmir_v14i5e124_app8.pdf]

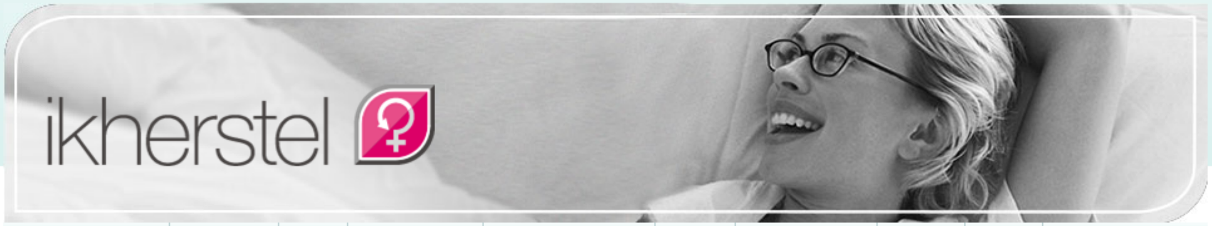

Informatie over de film

Deze film is gemaakt om inzicht te geven in de valkuilen en tips aan te dragen voor het oppakken van (werk) activiteiten na een gynaecologische operatie. De inhoud is gebaseerd op ervaringen van patiënten die reeds een gynaecologische operatie hebben ondergaan. In de film spelen twee verhaallijnen. Eén waarin het herstel en terugkeer naar werk goed verloopt en één waarin dit niet goed verloopt. De nadruk wordt steeds gelegd op factoren die het herstel en terugkeer naar werk positief of negatief kunnen beïnvloeden. Deze factoren zijn soms uitvergroot om het contrast te scheppen, maar komen in de realiteit wel voor en zijn hierdoor zinvol om bij stil te staan.
De gehele film duurt ongeveer 9 minuten. Voor het gemak is de film opgedeeld in drie hoofdstukken; 'Voor de operatie', 'Na de operatie' en '5 maanden later' zodat u niet genoodzaakt bent de gehele film te bekijken.

Trailer
Samenvatting

Deel 1
Voor de operatie

Deel 2
Na de operatie

Deel 3
Vijf maanden later

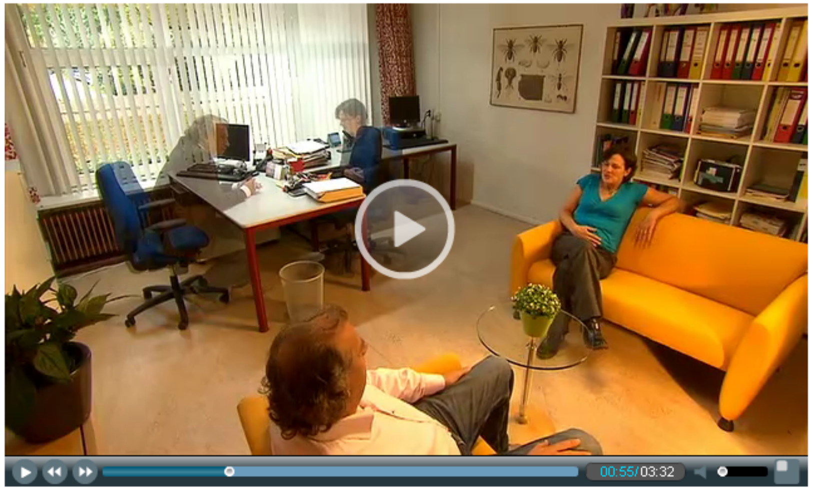

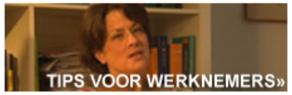

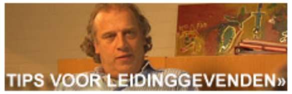

Copyright © 2009: ikherstel.nl
