# Supplementary material for: eHealth Program to Empower Patients in Returning to Normal Activities and Work After Gynecological Surgery: Intervention Mapping as a Useful Method for Development
Source: J Med Internet Res. 2012 Oct 19;14(5):e124. doi: 10.2196/jmir.1915 (PMC3510728; doi:10.2196/jmir.1915)
Supplement: Supplementary file 9 [file jmir_v14i5e124_app9.pdf]

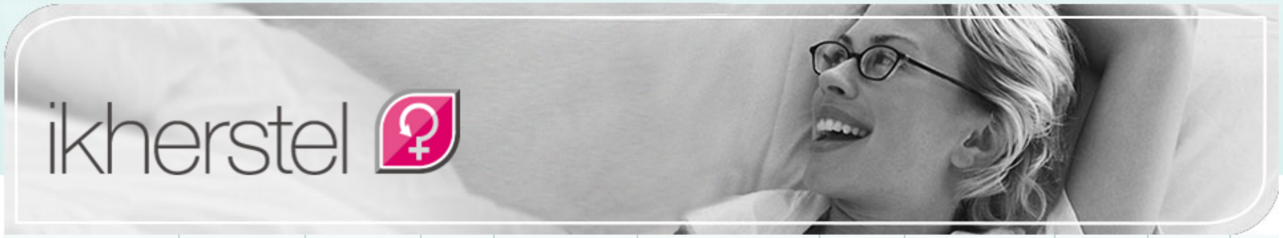

Richtlijnen

Op deze pagina vindt u per ingreep richtlijnen betreffende het hervatten van activiteiten, casuïstiek, achtergrondinformatie en factoren die het herstel kunnen beïnvloeden.

Laparoscopische adnexchirurgie

- Richtlijn ➔
  - Samenvatting richtlijn in tabelvorm
  - Samenvatting richtlijn in tekstvorm
  - Gedetailleerd overzicht voor het hervatten van activiteiten
  - Bijlage 1: definitie en gradering van de gebruikte items in het gedetailleerde overzicht
- Casuïstiek
- Achtergrondinformatie
- Medische factoren die het herstel beïnvloeden (nog niet beschikbaar)
- Niet-medische factoren die het herstel beïnvloeden (nog niet beschikbaar)

Laparoscopische Supracervicale Hysterectomie (LSH)

Totale Laparoscopische Hysterectomie (TLH)

Vaginale Uterus Extirpatie (VUE)

Abdominale Uterus Extirpatie (AUE)

Copyright © 2009: ikherstel.nl
