# Supplementary material for: eHealth Program to Empower Patients in Returning to Normal Activities and Work After Gynecological Surgery: Intervention Mapping as a Useful Method for Development
Source: J Med Internet Res. 2012 Oct 19;14(5):e124. doi: 10.2196/jmir.1915 (PMC3510728; doi:10.2196/jmir.1915)
Supplement: Supplementary file 10 [file jmir_v14i5e124_app10.pdf]

ikherstel

Definitief advies prive

Gebaseerd op operatiedatum: 11-11-2010

Uw operatie: TLH = Baarmoeder verwijdering via een kijkbuisoperatie

De gegeven adviezen geven aan wanneer de betreffende handelingen en activiteiten na een TLH operatie, door een expertgroep van artsen medisch gezien weer mogelijk worden geacht. Uw behandelend gynaecoloog heeft het schema (indien nodig) naar aanleiding van de operatie aangepast. De adviezen houden echter geen rekening met individuele beperkingen waardoor u bepaalde handelingen misschien niet mag verrichten. Raadpleeg bij twijfel uw behandelend gynaecoloog. Waar u in het document leest **'toegestaan vanaf'** betekent dit: **'mag medisch gezien uitgevoerd worden vanaf'**

Aantal dagen na de operatie	0	2	4	7	14	21	28	42
**Tillen of dragen**								
1 kg	*toegestaan vanaf 11-11-2010*							
5 kg		toegestaan vanaf	*15-11-2010*					
10 kg			toegestaan vanaf	*18-11-2010*				
15 kg				toegestaan vanaf	*25-11-2010*			
**Een aaneengesloten periode lopen**								
Minder dan 5 minuten	*toegestaan vanaf 11-11-2010*							
Ongeveer 15 minuten		toegestaan vanaf	*15-11-2010*					
Ongeveer 30 minuten			toegestaan vanaf	*18-11-2010*				
Ongeveer 1 uur				toegestaan vanaf	*25-11-2010*			
**Totale duur lopen per dag**								
Minder dan 30 minuten	*toegestaan vanaf 11-11-2010*							
Ongeveer 1 uur			toegestaan vanaf	*18-11-2010*				
Ongeveer 4 uur				toegestaan vanaf	*25-11-2010*			
Gehele dag (gedurende ± 8 uur)					toegestaan vanaf	*02-12-2010*		
**Frequent traplopen (ong. 5x / uur)**								
Ik moet één trap op- en aflopen			toegestaan vanaf	*18-11-2010*				
**Frequent knielen of hurken (ong. 10x per uur)**			toegestaan vanaf	*18-11-2010*				
**Stofzuigen**				toegestaan vanaf	*25-11-2010*			
**Fietsen**				toegestaan vanaf	*25-11-2010*			
**Autorijden**			toegestaan vanaf	*18-11-2010*				
(controleer ook altijd bij uw verzekering vanaf wanneer u verzekerd bent bij schade)								
**Een aaneengesloten periode zitten**								
Minder dan 15 minuten	*toegestaan vanaf 11-11-2010*							
Ongeveer 30 minuten	toegestaan vanaf	*13-11-2010*						
Ongeveer 1 uur	toegestaan vanaf	*13-11-2010*						
Ongeveer 2 uur		toegestaan vanaf	*15-11-2010*					
**Totale duur zitten per dag**								
Minder dan 4 uur	*toegestaan vanaf 11-11-2010*							
Ongeveer 4 uur			toegestaan vanaf	*18-11-2010*				
6 tot 8 uur			toegestaan vanaf	*18-11-2010*				
Gehele dag (gedurende ong. 8 uur)				toegestaan vanaf	*25-11-2010*			
**Een aaneengesloten periode staan**								
Minder dan 5 minuten	*toegestaan vanaf 11-11-2010*							
Ongeveer 15 minuten	toegestaan vanaf	*13-11-2010*						
Ongeveer 30 minuten			toegestaan vanaf	*18-11-2010*				
Ongeveer 1 uur				toegestaan vanaf	*25-11-2010*			
**Totale duur staan per dag**								
Minder dan 30 minuten	*toegestaan vanaf 11-11-2010*							
Ongeveer 1 uur			toegestaan vanaf	*18-11-2010*				
Ongeveer 4 uur				toegestaan vanaf	*25-11-2010*			
Gehele dag					toegestaan vanaf	*02-12-2010*		

Copyright © 2009: ikherstel.nl
